# Human Supernumerary Teeth-Derived Apical Papillary Stem Cells Possess Preferable Characteristics and Efficacy on Hepatic Fibrosis in Mice

**DOI:** 10.1155/2020/6489396

**Published:** 2020-01-30

**Authors:** Jun Yao, Nan Chen, Xiaojing Wang, Leisheng Zhang, Jiali Huo, Ying Chi, Zongjin Li, Zhongchao Han

**Affiliations:** ^1^School and Hospital of Stomatology, Fujian Medical University, Fuzhou, China; ^2^The Postdoctoral Research Station of Medicine College, Nankai University, Tianjin, China; ^3^Precision Medicine Division, Health-Biotech (Tianjin) Stem Cell Research Institute Co., Ltd., Tianjin 301700, China; ^4^State Key Laboratory of Experimental Hematology and Institute of Hematology & Blood Diseases Hospital, Chinese Academy of Medical Sciences & Peking Union Medical College, Tianjin, China

## Abstract

Dental tissue has been acknowledged as an advantaged source for high-quality dental pulp stem cell (DPSC) preparation. However, despite the accomplishment of the separation of DPSCs from permanent teeth and supernumerary teeth, the deficiency of rigorous and systematic clarification on the signatures and efficacy will hinder their prospects in regenerative medicine. In this study, we primitively isolated permanent teeth-derived DPSCs and supernumerary teeth-derived apical papillary stem cells (SCAP-Ss) with parental consent. Immunophenotype of DPSCs and SCAP-Ss was determined by a flow cytometry assay, and the cell viability was verified by multidimensional detections including cell proliferation, cell cycle, apoptosis, and senescence. The migration and clonogenic capacity were examined by a wound healing test and crystal violet staining, respectively. The multilineage differentiation potential was quantitated by utilizing Oil Red O staining and Alizarin Red staining, together with real-time PCR analysis. The efficacy on a mouse hepatic fibrosis model was evaluated by using histologic sections and liver function tests. Herein, we showed that SCAP-Ss exhibited comparable immunophenotype and adipogenic differentiation capacity as DPSCs. However, different from DPSCs, SCAP-Ss exhibited superiority in cell viability and osteogenic differentiation. Simultaneously, injection of DPSCs and SCAP-Ss significantly reduced inflammatory infiltration, enhanced liver-associated gene expression, and finally relieved symptoms of hepatic fibrosis. In conclusion, SCAP-Ss possess preferable characteristics and efficacy on hepatic fibrosis in mice. Our findings suggest that SCAP-Ss are an easily accessible postnatal stem cell source with multifaceted characteristics for regenerative medicine.

## 1. Introduction

Mesenchymal stem/stromal cells (MSCs) are acknowledged as a heterogeneous population with self-renewal and multilineage differentiation potential [1–3]. Owing to the unique hematopoietic-supporting and immunosuppressive properties, MSCs have been demonstrated as a key component of the microenvironment [4–6]. Originally, Friedenstein and his colleagues firstly isolated and identified MSCs from bone marrow in the 1960s [7]. Thereafter, MSCs were prepared from various tissues such as adipose, synovium, anadesma, dental pulp, placenta, and umbilical cord [3, 4, 8]. To date, longitudinal studies have illuminated the multidimensional signatures both at the cellular and molecular levels [3]. Moreover, an increasing number of preclinical and clinical studies are focused on the efficacy of MSCs in diversiform disease therapy, such as leukemia, osteoarthritis, hepatopathy, and diabetes [4, 9, 10]. Of them, bone marrow-derived MSCs (BM-MSCs) are the most commonly used sources in clinical trials [2, 3]. However, BM-MSCs have shortcomings such as invasiveness, long-term *in vitro* proliferation, and donor-specific variability in quality, together with pathogenic and ethical risks as well [2]. Hence, to better satisfy the clinical demands, alternative sources of MSCs become an urgent need [8].

To data, dental tissues including primary incisors, permanent teeth, and supernumerary teeth have attracted extensive attention as an easily accessible and noninvasive postnatal source of high-quality stem cells for tissue engineering applications [11, 12]. Interestingly, the dental tissue-derived cells share similarities in gene expression profile and multilineage differentiation capability to MSCs [13]. In the year of 2000, dental pulp stem cells (DPSCs) were firstly separated from permanent third molar teeth of different sections followed by other sections of oral parts including dental pulp, periodontal ligament, alveolar bone, gingiva, and dental follicle [11, 13, 14]. Recently, isolation and characterization of DPSCs from a “discarded” supernumerary tooth were primarily achieved by Huang and his colleagues [15]. Meanwhile, numerous investigators have also proactively explored the efficacy of these advantaged stem cells in various systemic disease treatment, including diabetes, muscular dystrophy, ischemic stroke, Alzheimer's disease, and eye disease [16, 17]. Unexpectedly, by practicing comparative analysis, Lee et al. and Seo et al. recently reported other subtypes of stem cells from human exfoliated deciduous teeth (SHED) and periodontal ligament stem cells (PDLSCs), which were distinguished from DPSCs, respectively [18, 19]. Similarly, to our knowledge, very limited studies have reported the stem cells from apical papilla of human supernumerary teeth (SCAP-Ss) and let alone the systematic evaluation of their signatures and efficacy in hepatic fibrosis [15].

In this study, we reported the isolation and identification of the abovementioned SCAP-Ss. Different from the supernumerary teeth-derived DPSCs, the SCAP-Ss possess preferable characteristics confirmed by multifaceted *in vitro* and *in vivo* analyses. Dramatically, the SCAP-Ss exhibited indiscriminate efficacy on hepatic fibrosis in mice. Taken together, we originally isolated and systematically evaluated SCAP-Ss as a unique alternative source of MSCs for future applications in regenerative medicine.

## 2. Materials and Methods

### 2.1. Stem Cell Culture and Passage

The SCAP-Ss and DPSCs were isolated from supernumerary teeth and permanent teeth of different patients (4–25 years old) according to the ethical committee of Fujian Medicine University, respectively (FYKLLSC-201921). In detail, the traditionally well-described DPSCs were isolated from the dental pulp cavity while the newly identified SCAP-Ss were derived from the apical papillary section of supernumerary teeth, which were structurally separated from the DPSCs and easily being distinguished by the dentist. The two stem cells at passages 3–8 were cultured and passaged as reported [13]. Briefly, the two types of stem cells were maintained in DMEM/F12 basal medium supplemented with 1% NEAA (Gibco), 1% L-glutamine (Gibco), 1% Penicillin and streptomycin (ThermoFisher), 10% fetal bovine serum (Australia), 4 ng/ml EGF (PeproTECH), and 4 ng/ml bFGF (PeproTECH). When the SCAP-Ss or DPSCs reached 80% confluency, cells were dissociated with 0.05% Trypsin-EDTA at 37°C for 5 min. After that, the stem cells were collected by centrifugation at 300 × g for 5 min at room temperature. After discarding the supernatant, the stem cells were resuspended and seeded in the abovementioned culture medium at 37°C, 5% CO_2_ as we recently reported [2, 3, 8].

### 2.2. Flow Cytometry Assay

A flow cytometry (FCM) assay was conducted as we recently reported with several modifications [2, 3, 20]. Briefly, SCAP-Ss and DPSCs were dissociated into single cells by 0.05% Trypsin-EDTA (Gibco) and labelled with the indicated antibodies against CD31, CD34, CD44, CD45, CD73, CD90, CD105, HLA-DR, Annexin V, 7-AAD, or PI, in 0.2% BSA for 30 min in the dark. Then, the cells were washed with 1x PBS twice and analysed by FACSCanto II (BD Biosciences). The FCM data were analysed with FlowJo 7.0 (Ashland). The antibodies were listed in Supplementary Table 1.

### 2.3. Cell Proliferation Detection

Cell proliferation was determined by using a cell counting kit-8 (CCK-8, Yeasen) reagent according to the manufacturer's instruction. 1 × 10^4^ SCAP-Ss and DPSCs were seeded in a 96-well plate (100 *μ*l/well) with 5 replications for each time point (d0-d7) at 37°C, 5% CO_2_. Then, the SCAP-Ss and DPSCs were incubated with 10 *μ*l CCK-8 and measured by using a multiscan spectrum (ThermoFisher) under absorbance at A450.

### 2.4. Cell Apoptosis Detection

The apoptosis in SCAP-Ss and DPSCs was detected by using the Annexin V-FITC/7-AAD Apoptosis Detection Kit (Yeasen). The SCAP-Ss and DPSCs were dissociated into single cells and washed with 1x PBS twice and resuspended with 100 *μ*l 1x binding buffer. Then, the cells were incubated with 5 *μ*l Annexin V-FITC and 10 *μ*l 7-AAD staining solution for 15 min at RT. Finally, the cells were washed with 400 *μ*l 1x binding buffer and detected by FACSCanto II (BD Biosciences).

### 2.5. Immunofluorescence Staining

The expression level of *β*-GAL was detected by using immunofluorescence staining according to the manufacturer's instruction as we described recently [8, 21]. Briefly, the SCAP-Ss and DPSCs were washed with 1x PBS twice and fixed with 4% formaldehyde for 30 min. Then, the indicated cells were blocked with blocking buffer for 1 hr at room temperature (RT). After that, the SCAP-Ss and DPSCs were permeabilized with 0.2% Triton™ X-100 and stained with rabbit anti-human *β*-GAL and with donkey anti-rabbit Alexa Fluor 488-conjugated secondary antibody, respectively. DAPI was used for nucleus staining.

### 2.6. Karyotypic Analysis

The karyotypic analysis was performed to monitor the stability of chromosomes in SCAP-Ss and DPSCs at passage 10 using a G-banding technique as we recently reported [3, 8]. Then, the karyotype of SCAP-Ss and DPSCs was captured by using an Olympus DA71 microscope (Tokyo, Japan) with 200x magnification.

### 2.7. *In Vitro* Wound Healing Assay

SCAP-Ss and DPSCs were seeded in a 6-well plate as previously reported [22]. When the SCAP-Ss or DPSCs reached 80% confluency, the stem cell layers were scratched with 200 *μ*l tips and captured by using an Olympus DA71 microscope (Tokyo, Japan) at the indicated time points.

### 2.8. Colony Forming Unit-Fibroblast (CFU-F) Assay

A standard CFU-F assay of SCAP-Ss and DPSCs was performed as we recently reported [2, 3]. In brief, the stem cells were detached with 0.25% trypsin/EDTA and seeded at a density of 100 cells per 10 cm dish in the aforementioned medium. After 14 days, the colonies were fixed with 4% PFA (Gibco) and dyed with 0.2% crystal violet solution (Solarbio). The colonies with more than 30 cells were calculated.

### 2.9. Multilineage Differentiation Analysis of SCAP-Ss and DPSCs

SCAP-Ss and DPSCs at indicated passages were seeded at a density of 5 × 10^4^/cm^2^ in the aforementioned culture medium as reported [2, 8]. When cells reached 80% confluency, the medium was changed into adipogenic differentiation medium (MesenCult Adipogenic Differentiation Kit, Stem Cell Technologies) or osteogenic differentiation medium (MesenCult Osteogenic Differentiation Kit, Stem Cell Technologies). The differentiation medium was changed every 3.5 days as we described previously [2, 3, 23]. 21 days later, the SCAP-S and DPSC-derived cells were stained with Oil Red O or Alizarin Red staining buffer, respectively. Then, the cells were photographed with a Nikon Eclipse Ti-U microscope (Nikon, Tokyo, Japan).

### 2.10. Quantitative Real-Time PCR Assay

A quantitative real-time PCR assay (qRT-PCR) was performed as we previously reported [21, 23, 24]. As to stem cells, the SCAP-Ss and DPSCs at indicated time points were washed with 1x PBS twice. As to liver tissue from mice, we used a part of the liver tissue from the indicated mice (NC, CCl_4_, CCl_4_+SCAP-S, and CCl_4_+DPSC) for total RNA extraction after treatments with liquid nitrogen and mechanical lapping. Then, total RNA of stem cells and liver tissue was collected by using a TRIzol reagent (ThermoFisher) according to the manufacturer's instruction. Then, the cDNA was synthesized by utilizing the TransScript Fly First-Strand cDNA Synthesis SuperMix (TransGen Biotech, China). qRT-PCR was performed with the ABI PRISM 7900 (Applied Biosystems) and the SYBR Green PCR Master Mix kit (Qiagen). The primer sequences are available in Supplementary Table 2.

### 2.11. Hepatic Fibrosis Model in Mice

Six-week-old NOD-SCID mice were purchased from Fujian Medical University. The mice were kept in SPF laboratory and used for experiments under the protocols approved by the animal experiment ethical committee of Fujian Medical University (FYKDWLLSC-201912). To induce the hepatic fibrosis model, 40 *μ*l sterilized 20% CCl_4_ was intravenously injected into NOD-SCID mice for 8 weeks (once a week). Then, the mice were randomized into the control group (CCl_4_) and the experimental groups (CCl_4_+SCAP-S, CCl_4_+DPSC). Meanwhile, the NOD-SCID mice that received the same volume of 0.9% saline solution instead of 20% CCl_4_ were used as the negative control (NC). The mice in experimental groups were treated with 4 × 10^4^ SCAP-Ss or PPSCs through intravenous injection for 4 weeks (once a week). At the 13th week, all mice were euthanatized for pathologic or liver function analyses.

### 2.12. Histologic Section and Staining

Histologic section of the liver in mice was treated with hematoxylin-eosin (H&E) staining or mason staining as we described before [3, 25]. Then, the sections were observed under a Nikon Eclipse Ti-U microscope (Nikon, Tokyo, Japan).

### 2.13. Statistical Analysis

Statistical analysis in the study was performed by utilizing Prism 5 software (GraphPad, San Diego, USA) as we described [2, 21, 24, 25]. In brief, an unpaired *t*-test was conducted to analyse the data of two unpaired groups, and one-way ANOVA with Tukey's post hoc test was used to analyse the data of multiple unpaired groups. All the data were shown as the mean ± SE (*N* = 3 independent experiments); *p* < 0.05 were considered statistically significant (^∗^*p* < 0.05, ^∗∗^*p* < 0.01, and ^∗∗∗^*p* < 0.001; NS: not significant).

## 3. Results

### 3.1. Isolation and Immunophenotypic Identification of SCAP-Ss and DPSCs

**Figure 1:**
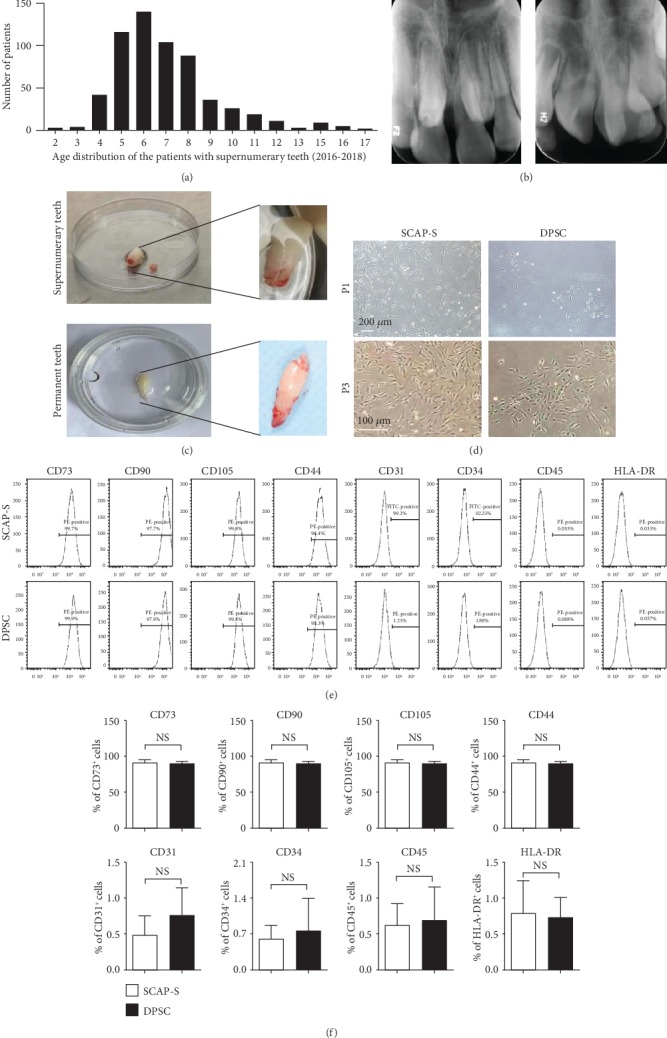
Isolation and phenotype of SCAP-Ss and DPSCs. (a) Age distribution of the patients with supernumerary teeth during the years of 2016–2018 at the dental clinic of the First Affiliated Hospital of Fujian Medical University. (b) The periapical radiography of the supernumerary teeth (mesiodens) and permanent teeth of the patients in this study. (c) The removed supernumerary tooth and permanent tooth in a 10 cm dish. (d) Phase contrast images of primary (upper panel) and passages (bottom panel) of SCAP-Ss and DPSCs derived from the supernumerary tooth and permanent tooth (P1, P3), respectively. Scale bar = 200*μ*m (upper panel) or scale bar = 100*μ*m (bottom panel). (e) Flow cytometry (FCM) analysis for surface markers (CD73, CD90, CD105, CD44, CD31, CD34, CD45, and HLA-DR) of SCAP-Ss and DPSCs in DMEM/F12 basal medium supplemented with 1% NEAA (Gibco), 1% L-glutamine (Gibco), 1% Penicillin and streptomycin (ThermoFisher), 10% fetal bovine serum, 4 ng/ml EGF, and 4 ng/ml bFGF. (f) Statistical analysis of FCM data in (e). All data were shown as the mean ± SEM (*N* = 3). NS: not significant.

Supernumerary teeth (mesiodens) are common symptoms of the teenagers at the dental clinic. During the year of 2016–2018, a total number of 608 patients with supernumerary teeth received treatment in our hospital (Figures 1(a) and 1(b)). To our knowledge, although DPSCs have been separated from permanent teeth for decades, very limited reports have been documented on deriving the high-quality SCAP-Ss from mesiodens and the systematic comparison between SCAP-Ss and DPSCs is largely lacking [15]. Herein, we primitively isolated the abovementioned stem cells from anonymized patients with the permission of the clinical research ethics committee as described previously [13] (Figures 1(c) and 1(d)). Strikingly, the primary and passages of SCAP-Ss showed spindle-like morphology with DPSCs but with larger quantity (Figure 1(d)). Then, we measured the expression of MSC-associated (CD73, CD90, CD105, and CD44), endothelial and hematopoietic-associated (CD31, CD34, and CD45), and immune-associated (HLA-DR) surface markers with the flow cytometry assay. Expectedly, both the SCAP-Ss and the DPSCs congruously expressed high levels of MSC-associated markers whereas mere expression of endothelial or hematopoietic-associated markers (Figures 1(e) and 1(f)). Taken together, we successfully obtained SCAP-Ss and DPSCs from supernumerary and permanent teeth, respectively.

### 3.2. SCAP-Ss Exhibit Superiority in Cell Viability over DPSCs

**Figure 2:**
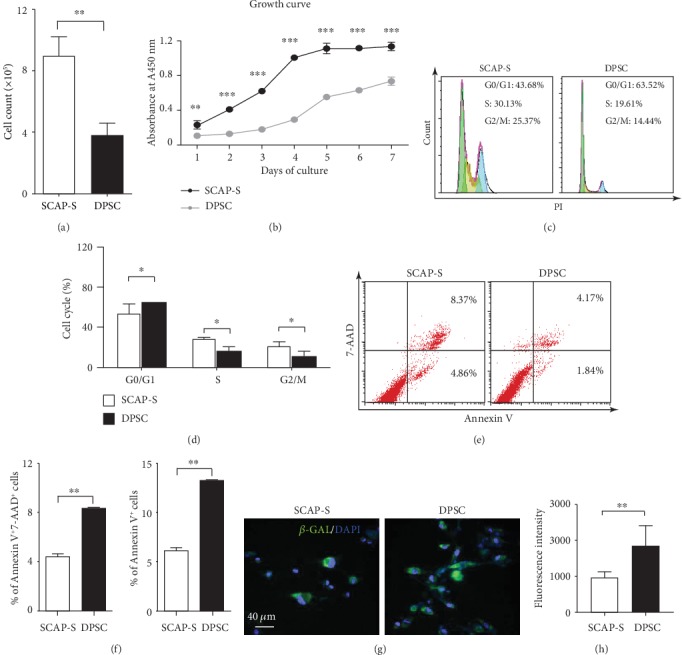
Cell viability analysis of SCAP-Ss and DPSCs. (a) The comparison of the total cell number between SCAP-Ss and DPSCs maintained in the abovementioned culture medium. All data were shown as the mean ± SEM (*N* = 3). ^∗∗^*p* < 0.01. (b) The growth curves of SCAP-Ss and DPSCs maintained in the abovementioned culture medium. All data were shown as the mean ± SEM (*N* = 3). ^∗∗^*p* < 0.01; ^∗∗∗^*p* < 0.001. (c) FCM analysis of the distribution of the sub-G0/G1, S, and G2/M phases of the cell cycle in SCAP-Ss and DPSCs. (d) Statistical analysis of FCM data in (c). All data were shown as the mean ± SEM (*N* = 3). ^∗^*p* < 0.05. (e) FCM analysis of the distribution of the SCAP-Ss and DPSCs with apoptotic or preapoptotic properties. The total stem cells were incubated with the 7-AAD and Annexin V antibodies. (f) Statistical analysis of FCM data in (e). All data were shown as the mean ± SEM (*N* = 3). ^∗∗^*p* < 0.01. (g) Immunofluorescent staining showed that the cultured SCAP-Ss and DPSCs with senescent characteristics were stained with *β*-GAL. DAPI was used for nucleus staining. Scale bar = 40*μ*m. (h) Statistical analysis of fluorescence intensity data in (g). All data were shown as the mean ± SEM (*N* = 3). ^∗∗^*p* < 0.01.

Having noticed the difference in the number of primarily isolated cells and passages of cultured cells (Figure 1(d)), we speculated that the cell viability of SCAP-Ss might be superior to that of DPSCs. For the purpose, we primitively conducted a population doubling assay and found that SCAP-Ss were approximately 2-fold over DPSCs, which was further confirmed by the growth curve (Figures 2(a) and 2(b)). Thereafter, with the aid of FCM analysis, we noticed the differences in the distribution of multiple cell cycle phases between SCAP-Ss and the control DPSCs (Figure 2(c)). In general, more cells were distributed in the S and G2/M phases of the cell cycle, which indicated an increasing proportion of division in SCAP-Ss (Figure 2(d)). On the contrary, a lower proportion of population with apoptotic or preapoptotic property in SCAP-Ss was identified by Annexin V and 7-AAD staining (Figures 2(e) and 2(f)). In addition, we also identified that a higher percentage of DPSCs was senescent compared with that of SCAP-Ss (Figures 2(g) and 2(h)). In summary, the prepared SCAP-Ss exhibited superiority in cell viability over DPSCs.

### 3.3. SCAP-Ss Showed Preferable Migration and Colony Forming Capacity over DPSCs

**Figure 3:**
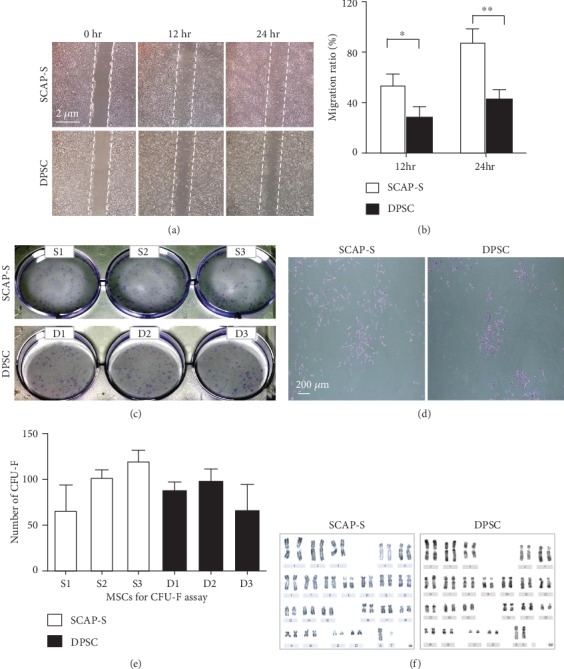
Migration, CFU-F formation, and karyotypic analysis of SCAP-Ss and DPSCs. (a) The comparison of migration capacity by the wound healing assay between SCAP-Ss and DPSCs cultured in the abovementioned culture medium. Scale bar = 2*μ*m. (b) Statistical analysis of wound healing data in (a). All data were shown as the mean ± SEM (*N* = 3). ^∗^*p* < 0.05; ^∗∗^*p* < 0.01; NS: not significant. (c) Phase contrast images of CFU-Fs formed by the seeded SCAP-Ss and DPSCs, respectively. (d) Typical CFU-Fs with more than 30 cells were dyed with crystal violet solution. Scale bar = 200*μ*m. (e) Statistical analysis of CFU-F data in (c). All data were shown as the mean ± SEM (*N* = 3). (f) Karyotypic analysis of SCAP-Ss and DPSCs with G-banded chromosome experiment.

To further dissect the potential distinctions in biological signatures, we subsequently examined the migration and clonogenic capacity of the aforementioned stem cells. By conducting wound healing analysis, we found that SCAP-Ss could transfer more easily and rapidly than DPSCs (Figures 3(a) and 3(b)). However, both of the two types of stem cells showed comparable typical colony forming capacity (Figures 3(c)–3(e)). In addition, with the aid of G-banded chromosome analysis, we verified that SCAP-Ss exhibited normal karyotype without gross abnormalities at the genomic level as DPSCs (Figure 3(f)).

### 3.4. SCAP-Ss Possess Favourable Osteogenic Differentiation Potential *In Vitro*

**Figure 4:**
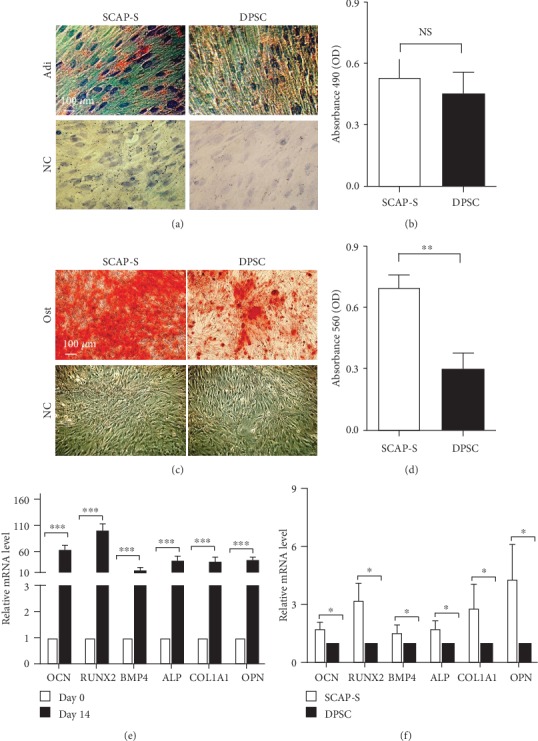
Multilineage differentiation analysis of SCAP-Ss and DPSCs. (a) Adipogenic differentiation potential of SCAP-S and DPSC-derived cells was identified by Oil Red O staining (scale bar = 100*μ*m). Undifferentiated SCAP-Ss and DPSCs were served as the negative control (NC). (b) Statistical analysis of Oil Red O staining data in (a). All data were shown as the mean ± SEM (*N* = 3). NS: not significant. (c) Osteogenic differentiation potential of SCAP-S and DPSC-derived cells was identified by Alizarin Red staining (scale bar = 100*μ*m). Undifferentiated SCAP-Ss and DPSCs were served as the negative control (NC). (d) Statistical analysis of Alizarin Red staining data in (c). All data were shown as the mean ± SEM (*N* = 3). ^∗∗^*p* < 0.01. (e) qRT-PCR analysis of the osteogenic markers (*OCN*, *RUNX2*, *BMP4*, *ALP*, *COL1A1*, and *OPN*) in undifferentiated SCAP-S (day 0) and SCAP-S-derived cells (day 14). All data were shown as the mean ± SEM (*N* = 3). ^∗∗∗^*p* < 0.001. All values are normalized to the day 0 group (=1). (f) qRT-PCR analysis of the abovementioned osteogenic markers in SCAP-S and DPSC-derived cells (day 14). All data were shown as the mean ± SEM (*N* = 3). ^∗^*p* < 0.05. All values are normalized to the DPSC group (=1).

Previous studies have indicated that both permanent teeth- and supernumerary teeth-derived DPSCs exhibited multidirectional differentiation potential analogously to mesenchymal stem cells (MSCs) [18]. Therefore, we assumed that the SCAP-Ss were intrinsically a subtype of MSC-like DPSCs. Thus, we took advantage of multilineage differentiation analyses to explore the potential differences. As shown by Oil Red O staining, comparable adipose droplets were generated from SCAP-Ss and DPSCs after 3 weeks of adipogenic differentiation (Figures 4(a) and 4(b)). By contrast, SCAP-S-derived cells showed favourable osteogenic differentiation potential than those in the DPSC group as shown by Alizarin Red staining (Figures 4(c) and 4(d)). Quantitative analysis of multiple osteogenic markers in DPSC-derived cells (day 14) and undifferentiated DPSCs (day 0), including *OCN*, *RUNX2*, *BMP4*, *ALP*, *COL1A1*, and *OPN*, by qRT-PCR further confirmed the cytomorphological analysis of osteogenic differentiation (Figure 4(e)). Different from the DPSC-derived cells, the expression levels of the indicated osteogenic markers in SCAP-S-derived cells were much higher (Figure 4(f)). Taken together, these data further reinforced the conclusion that SCAP-Ss have preferable osteogenic differentiation potential over DPSCs both at the cellular and molecular levels.

### 3.5. Treatment with SCAP-S Effectively Alleviates Hepatic Fibrosis *In Vivo*

**Figure 5:**
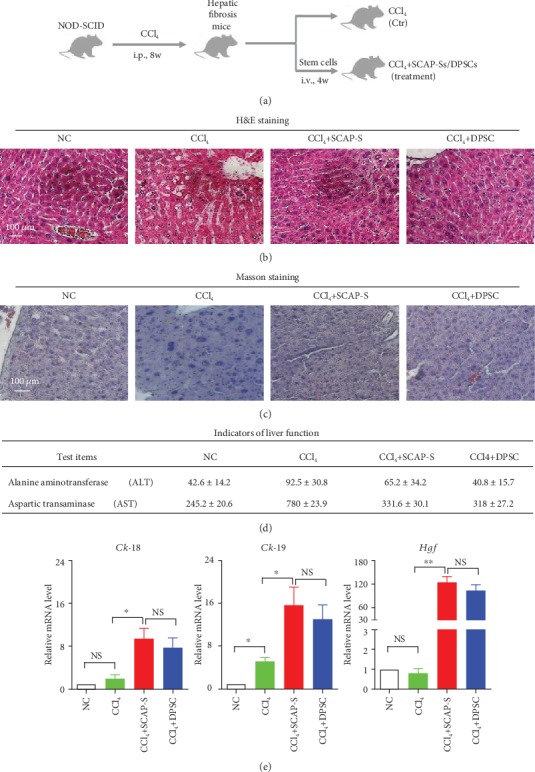
Preclinical therapeutic effect of SCAP-Ss and DPSCs on mice with hepatic fibrosis. (a) Schematic of the process of inducing the hepatic fibrosis model and the efficacy of SCAP-Ss and DPSCs on mice. (b) Histopathologic analysis of liver tissues of mice (NC, CCl_4_, CCl_4_+SCAP-Ss, and CCl_4_+DPSCs) at week 12 by hematoxylin and eosin (H&E) staining. NC: negative control. Scale bar = 100*μ*m. (c) Histopathologic analysis of the indicated liver tissues of mice at week 12 by Masson staining. Scale bar = 100*μ*m. (d) Indicators of liver function including alanine aminotransferase (ALT) and aspartic transaminase (AST) in the indicated mice at week 12. Briefly, the serum was enriched into a 1.5 ml microanticoagulant tube from peripheral blood of mice by centrifugation at 300 × g for 5 min in the indicated groups (NC, CCl_4_, CCl_4_+SCAP-Ss, and CCl_4_+DPSCs). Then, the ALT value and AST value in the serum were qualified with the commercial automatic biochemical analyser according to the instructions. All data were shown as the mean ± SEM (*N* = 3). (e) qRT-PCR analysis of the liver reconstruction-associated genes (*Ck-18*, *Ck-19*, and *Hgf*) in the indicated mice at week 12 as described in Materials and Methods. All data were shown as the mean ± SEM (*N* = 3). ^∗^*p* < 0.05; NS: not significant. All values are normalized to the NC group (=1).

Recently, we and other investigators have demonstrated that adult tissue-derived MSCs exhibit favourable therapeutic effect on hepatic fibrosis [16]. Herein, we took advantage of the moue model to assess whether hepatic fibrosis in mice could be effectively ameliorated by SCAP-S or DPSC transplantation. As shown in Figure 5(a), to induce the hepatic fibrosis model, 40 *μ*l sterilized CCl_4_ was intravenously injected into NOD-SCID mice for 8 weeks. After that, the mice were randomized into the control group (CCl_4_) with saline injection and the experimental groups with 2 × 10^5^ stem cell (CCl_4_+SCAP-S, CCl_4_+DPSC) treatment for four times (once a week). Finally, at week 13, all mice were euthanatized for pathologic or liver function analyses. Compared to the control group, mice that received systemic infusion of SCAP-Ss or DPSCs exhibited reduced inflammatory infiltration and pathological changes of liver tissue, which were confirmed by the histologic sections with H&E staining and Masson staining (Figures 5(b) and 5(c)). Consistent with the pathological indicators, the levels of alanine aminotransferase (ALT) and aspartic transaminase (AST) in the serum isolated from peripheral blood were significantly ameliorated in mice with SCAP-Ss or DPSC treatment (Figure 5(d)). Furthermore, the expression of liver reconstruction-associated genes, including *Ck-18*, *Ck-19*, and *Hgf*, was further increased in the experimental groups, which indicated the alleviated fibrosis by both SCAP-Ss and DPSCs.

## 4. Discussion

Longitudinal studies have demonstrated dental tissue as an easily accessible and noninvasively postnatal source of high-quality dental pulp stem cells (DPSCs) [11]. Of them, primary incisors, permanent third molar teeth, and deciduous teeth were considered the most common source of DPSCs with extensive prospects for regenerative medicine [16]. By contrast, very limited progress has been made on stem cells from the “discarded” apical papilla of human supernumerary teeth (SCAP-Ss) [15]. Above all, the systematic comparison of SCAP-Ss with DPSCs is largely obscure as well. Herein, we originally and resoundingly isolated and identified SCAP-Ss as an alternative source of MSCs from the “discarded” supernumerary teeth, which showed preferable characteristics over DPSCs from permanent teeth both *in vitro* and *in vivo*. For instance, unlike DPSCs, these SCAP-Ss exhibited enhanced cell viability including superior proliferation and survivability, together with remarkable migration and osteogenic differentiation potential. What is more, the therapeutic effect of SCAP-Ss and DPSCs on hepatic fibrosis in mice was investigated for the first time. Compared with the control group (CCl_4_), intravenous injection of SCAP-Ss significantly alleviated the pathological status of hepatic fibrosis by ameliorating the inflammatory infiltration, indicators of liver function (ALT, AST), and liver reconstruction-associated genes (*Ck-18*, *Ck-19*, and *Hgf*).

For long stretches, no report has been documented on isolating DPSCs from other tooth types including the supernumerary tooth. Until the year of 2007, Huang et al. firstly identified DPSCs from the supernumerary tooth [15]. However, in this study, the identified SCAP-Ss were isolated from the apical papilla of human supernumerary teeth, which was distinguished from the reported DPSCs. Recently, Lee and his colleagues have emphasized one possibility on other stem cells from human exfoliated deciduous teeth (SHEDs), which are identified as a population distinct from DPSCs [18]. Therefore, in conjunction with the aforementioned distinctions with DPSCs in biological signatures, we speculated that the SCAP-Ss in our studies were much inclined to an alternative new source of MSCs or neural crest-derived stem cells as we recently reported [2, 3]. Herein, our study originally offers new references for systematically investigating and comparing the characteristics of SCAP-Ss with DPSCs. In addition, during tooth germ development, there is usually an extra tooth germ in teenagers besides the permanent teeth, especially during the period of permanent teeth replacing the deciduous teeth [26, 27]. To our knowledge, the eruption potential mainly relates to personal development and hereditary factors, but we do agree with the speculation that the eruption potential also relates to the best response of the pulp cells. For instance, the supernumerary teeth-derived apical papillary stem cells (SCAP-Ss) in this study exhibited superiority in cell viability and osteogenic differentiation, which might help accelerate and enhance the abnormal tooth germ formation in children. Therefore, along with the aforementioned similarities and distinctions in biological signatures between SCAP-Ss and DPSCs at the cellular level, it is of equal importance to further systematically and thoroughly dissect the potential characteristics at the molecular level as we recently reported on UC-MSCs [3]. On the one hand, although we have indicated the preferable osteogenic differentiation capacity of SCAP-Ss over DPSCs, the underlying mechanism including the pivotal genes and signalling pathway, together with the interaction networks, orchestrates that the cytomorphology and biological functions are largely inaccessible and dauntingly complex. On the other hand, the meticulous illumination of the molecular genetic characteristics will be beneficial to clarify the detailed genetic alterations in adolescents with supernumerary teeth as well.

To date, DPSCs together with other dental tissue-derived cells, including SCAP-Ss, SHEDs, and PDLSCs, have been demonstrated as preferable alternatives of BM-MSCs for future regenerative medicine [16, 17]. For instance, the large quantity of the “discarded” supernumerary teeth in children and adolescents supplies advantaged new sources for SCAP-S isolation [26, 27]. Above all, the reported SCAP-Ss in this study exhibit multidimensional advantages such as the cell number and viability, together with no ethical risk. Simultaneously, the *in vivo* transplantation results have also enlightened the prospect of SCAP-Ss as an effective therapeutic intervention for liverish patients with multiple manifestation of metabolic syndrome with poor disability and outcome including cirrhosis and fibrosis. However, before large-scale clinical applications, systematic and detailed comparison both at the cellular and molecular levels is a prerequisite to explore the potential issues on safety, effectiveness, and repeatability like BM-MSCs.

## 5. Conclusions

In the present study, we have isolated and identified a new source of stem cells from the apical papilla of human supernumerary teeth (SCAP-S). Compared to the dental pulp stem cells (DPSC), SCAP-S show superiority in biological signatures including higher proliferation capacity, cell cycling, and multidifferentiation potential and lower rates of apoptosis and senescence but with comparable therapeutic effect as DPSC.

## Data Availability

The data used to support the findings of this study are available from the corresponding author upon request.

## Supplementary Materials

Supplementary Materials Supplementary Table 1: the antibodies for flow cytometry analysis. Supplementary Table 2: the primer sequences of indicated genes for quantitative RT-PCR analysis.
