# Supplementary material for: Human Supernumerary Teeth-Derived Apical Papillary Stem Cells Possess Preferable Characteristics and Efficacy on Hepatic Fibrosis in Mice
Source: Stem Cells Int. 2020 Jan 30;2020:6489396. doi: 10.1155/2020/6489396 (PMC7204141; doi:10.1155/2020/6489396)
Supplement: Supplementary Materials — Supplementary Table 1: the antibodies for flow cytometry analysis. Supplementary Table 2: the primer sequences of indicated genes for quantitative RT-PCR analysis. [file 6489396.f1.pdf]

Human Supernumerary Teeth-derived Apical Papillary Stem Cells Possess Preferable Characteristics and Efficacy on Hepatic Fibrosis in Mice

Jun Yao,[1] Nan Chen,[1] Xiaojing Wang, [1] Leisheng Zhang,[2,3,4,*] Jiali Huo,[4] Ying Chi,[4] Zongjin Li,[3] and Zhongchao Han[4]

[1] School and Hospital of Stomatology, Fujian Medical University, Fuzhou, China.

[2] The Postdoctoral Research Station of Medicine College, Nankai University, Tianjin, China.

[3] Precision Medicine Division, Health-Biotech (Tianjin) Stem Cell Research Institute Co., Ltd., Tianjin, 301700, China.

[4] State Key Laboratory of Experimental Hematology and Institute of Hematology & Blood Diseases Hospital, Chinese Academy of Medical Sciences & Peking Union Medical College, Tianjin, China.

E-mail Address of all the listed co-authors (Not all the co-authors have full institutional mailing addresses):

Jun Yao, dentyao@163.com;

Nan Chen, 398577287@qq.com;

Xiaojing Wang, wangxj2925@foxmail.com;

Leisheng Zhang, zhangleisheng@health-biotech.com;

Jiali Huo, huojiali1990@163.com;

Ying Chi, chiying@ihcams.ac.cn;

Zongjin Li, zongjinli@nankai.edu.cn;

Zhongchao Han, hanzhongchao@health-biotech.com.

[*] Corresponding author: Leisheng Zhang

Correspondence should be addressed to Leisheng Zhang; zhangleisheng@health-biotech.com

Supplementary Materials

Supplementary Table 1: The antibodies for flow cytometry analysis.

Antioody	Cat. NO.	Source
Anti-human CD31-PE	555446	BD Pharmigen
Anti- human CD34-APC	555824	BD Pharmigen
Anti-human CD44-PE	550989	BD Pharmigen
Anti- human CD45-PE	560975	BD Pharmigen
Anti- human CD73-PE	550257	BD Pharmigen
Anti- human CD90-APC	328113	BioLegend
Anti- human CD105-APC	323208	BioLegend
Anti-human HLA-DR-Cy7	25-9956-71	eBioscience

Supplementary Table 2: The primer sequences of indicated genes for quantitative RT-PCR analysis.

Gene	Forward Primer	Reverse Primer
OCN	CACTCCTCGCCCTATTGGC	CCCTCCTGCTTGGACACAAAG
RUNX2	CTCACTACCACACCTACCTG	TCAATATGGTCGCCAAACAGATTC
BMP4	ATGATTCCTGGTAACCGAATGC	CCCCGTCTCAGGTATCAAACT
ALP	GAGATGTTGTCCTGACACTTGTG	AGGCTTCCTCCTTGTTGGGT
COL1A1	GAGGGCCAAGACGAAGACATC	CAGATCACGTCATCGCACAAC
OPN	CTCCATTGACTCGAACGACTC	CAGGTCTGCGAAACTTCTTAGAT
Ck-18	CAGCCAGCGTCTATGCAGG	CCTTCTCGGTCTGGATTCCAC
Ck19	GTTCAGTACGCATTGGGTCAG	GAGGACGAGGTCACGAAGC
Hgf	ACTTCTGCCGGTCCTGTTG	CCCCTGTTCCTGATACACCT
